# Preschool Temperament as a Factor of Risk and Protection for Later Childhood Psychopathology

**DOI:** 10.3389/fpsyt.2022.803959

**Published:** 2022-06-01

**Authors:** Frank W. Paulus, Eva Möhler, Lisa Festag, Jens Joas

**Affiliations:** Department of Child and Adolescent Psychiatry, Saarland University Hospital, Homburg, Germany

**Keywords:** temperament, risk factor, resilience factor, mental health, psychopathology

## Abstract

**Background:**

Temperament might be considered as a risk factor as well as a resilience factor for later externalizing and internalizing disorders. Therefore, this study examines different dimensions of temperament in preschool age with regard to their predictive value for psychopathology later in childhood.

**Methods:**

A total of 76 patients (63.2% male) were assessed in a special psychiatric consultation for preschool age at measuring point time t1 (*x* = 4.2) and measuring point time t2 (*x* = 9.2). At t1, the Integrative Child Temperament Inventory (ICTI) was used for assessment. At t2, parents completed the Strengths and Difficulties Questionnaire SDQ. Multiple regression analyses were used to test if the temperament factors of the ICTI predicted clinical abnormalities in the SDQ subscales or total difficulties score.

**Results:**

SDQ total difficulties score as an indicator of total psychiatric disturbance in childhood appears to be good predicted by the temperament factor frustration/anger. Sensory sensitivity in preschoolers serves as a risk factor for later emotional symptoms, whereas high activity levels appear to prevent later emotional symptoms. Behavioral inhibition appears to protect against hyperactivity/inattention.

**Conclusion:**

Our data suggests that preschool temperament contributes differently to the development of externalizing and internalizing problems in childhood. The temperament factor frustration/anger in preschool children might be a strong predictor of the general mental condition in childhood at nine years of age and can therefore be used as a target for prevention of psychopathology in children. On one hand, high sensory sensitivity can be a predictor to identify preschool children at risk for later emotional symptoms, on the other hand, activity level acts as a protective factor against later emotional symptoms. An increased level of behavioral inhibition might be protective against the development of hyperactivity/inattention symptoms. Overall, this study illustrates the complexity and ambiguity of temperament in child development.

## Introduction

### Definitions and Theories

Temperament conceptualizes individual differences in affective, motor, attentional, and sensory reactivity that have a biological basis and provide the foundation for later personality (1). Numerous approaches to child temperament have been proposed (2–6). Thomas and Chess (6) argued that “temperament can be equated with the term *behavioral style.*” Thus, it refers to the *how* rather than the *what* of behavior. In their longitudinal study, Thomas and Chess (7) identified three fundamental temperament types: the “difficult temperament,” the “easy temperament,” and the “slow-to-warm-up temperament.” Goldsmith and Campos (3) defined temperament as individual differences in the emotional domain, thereby including not only emotion but also emotion regulation. Rothbart and Derryberry (8) emphasized the role of neurobiological factors in child temperament. Following this statement, temperament involves constitutional differences in reactivity and self-regulation. While reactivity describes the biological arousability in response to changes in the environment, self-regulation describes processes that modulate this reactivity. On a behavioral level, self-regulation displays tendencies such as approach, avoidance, inhibition, and attentional self-regulation (8).

Summarizing and further developing these approaches, Zentner and Bates (9) proposed an integrative account of child temperament, according to which temperament comprises five components: frustration/anger, behavioral inhibition, activity, attention/persistence, and sensory sensitivity. Frustration/anger is defined as “negative affect in reaction to interruption of ongoing tasks or blocking of behaviors related to approach and goal attainment” [(9), p. 18]. Behavioral inhibition characterizes fear or distress responses that occur automatically in novel situations. Activity level refers to the amount of movement. Attention/persistence can also be subsumed under the term of effortful control, which refers to the “ability to inhibit a dominant response and/or activate a subdominant response, to play, and to detect errors” [(10), p. 129]. Finally, sensory sensitivity refers to the sensitivity to aversive stimuli as well as the ability to react to sensory stimuli of low stimulative value (9).

Although possible overlaps are not denied by the authors, they assume that these temperament dimensions are distinct and each of them is connected to different neural circuits.

Beyond this dimensional view of temperament, the authors also postulate a typological approach to the organization of temperament dimensions [(9), p. 23]. The three distinctive types can be described as the “undercontrolled child” (willful, restless, inattentive, impulsive), the “overcontrolled child” (shy, obedient, self-critical, liked by adults), and the “resilient child” (self-confident, able to concentrate, self-reliant and open). Considering the attributes (in brackets) related to these three types, it appears as though some temperament components are stronger associated than others. However, given the extent of possible linkages between the different components, the authors also point out that little research has been conducted in respect of moderation between the different components.

Based on their integrative account, Zentner and Bates (9) proposed the following criteria for child temperament:

• Temperament conceptualizes individual differences in affect, activity, attention and sensory sensitivity.
• Temperament is typically expressed in response intensities, latencies, durations, thresholds, and recovery times.
• Temperament develops during the first few years of life.
• Counterparts exist in primates as well as certain social mammals.
• Temperament is linked to biological mechanisms (e.g., neurochemical, neuroanatomical, genetic).
• Temperament is characterized as relatively enduring and as the foundation for later personality as well as psychopathological outcomes, such as externalizing or internalizing problems.

### Temperament and Later Psychopathology

Empirical evidence for the long-lasting stability in temperament and personality traits is provided by a longitudinal study by Caspi (11), who investigated cohorts of children ranging from 3 to 21 years. In this study, children were assigned to one of three temperament groups at age three: the well-adjusted group, the undercontrolled group and the inhibited group, which resemble the “easy temperament,” the “difficult temperament,” and the “slow-to-warm up temperament” proposed by Thomas and Chess (6), respectively. The former type is characterized by demonstrating self-control and the absence of upset when confronted with strangers or new situations; the middle is characterized by acting impulsive, restless, negativistic, distractible and liable in their emotional control; the latter is characterized by behaving socially reticent, fearful, and easily upset by strangers (6). These three temperament groups predicted adult personalities at the age of 21. Children who were classified as undercontrolled at age three were described as more impulsive and aggressive at age 18 and had more employment difficulties and interpersonal conflicts at age 21. On the other hand, children who were described as inhibited at age three were found to be more unassertive and depressed later on in life. Furthermore, they had lower levels of social support. Children, who were classified as well-adjusted at age three, were described as “normal, average young adults” [(11), p. 168].

#### Temperament and General Psychiatric Disturbance

While child temperament provides the foundation for later personality, it might also serve as a predictor of later psychopathology [e.g., (10)]. In a recent study, Morales et al. (12) examined whether infant temperament factors, assessed at four months of age, predict general psychopathology 7–12 years later. The authors found that higher motor activity longitudinally predicted general psychopathology. Thus, infant motor activity comprises a transdiagnostic risk factor for general psychopathology. Their finding is in line with previous work demonstrating an associative linkage between general psychopathology and parent-reported surgency, a temperament dimension that consists of activity level, high intensity pleasure, impulsivity, and shyness (13). In addition, Wlodarczyk et al. (14) found a significant relation between children’s difficult temperament and the probability of them having mental health disorders.

#### Temperament and Emotional Problems

There are divergent understandings of emotional symptoms. We use this term consistent with the description implied by Goodman (15). Considering the five items of the emotional subscale, they reflect anxiety and depressed symptoms. Furthermore, they best predict emotional disorders (16) and thus belong to the subordinate internalizing spectrum (17). Anxiety and mood disorders are common mental disorders among childhood and adolescents (18). The prevalence of anxiety disorders among preschool aged children can be estimated as 9.4% while the prevalence of emotional disorders among preschool aged children can be estimated as 10.5–14.9% (19). Among temperament factors, behavioral inhibition can be seen as a risk factor for childhood anxiety [e.g., (20)]. Behavioral inhibition has been described as signs of uncertainty and physiological arousal in reaction to novel objects, people, or events (21, 22). Specifically, behavioral inhibition provides an important predictor for social anxiety, rather than anxiety disorders in general. For example, in a longitudinal study by Hudson et al. (23), children at age four were classified as either behaviorally inhibited or behaviorally uninhibited. The authors found that behaviorally inhibited children were more likely to meet the criteria for social phobia at age six. Atypically high levels of depressive and anxious symptoms at preschool age may be predicted by a difficult temperament at 5 months of age (24). Furthermore, Schwartz et al. (25) found that adolescents who showed behavioral inhibition at age 2 were more likely to develop symptoms of social anxiety than uninhibited peers. Moreover, sensory sensitivity is associated with emotional problems, especially internalizing problems, such as anxiety, depression and withdrawal (26, 27).

#### Temperament and Conduct Problems

Conduct problems comprise symptoms of the diagnostic categories of oppositional defiant disorder (ODD) and conduct disorder (CD) (28). Several studies have linked several temperament factors to conduct problems in preschool age, school age and adolescence [for a review see (29)]. Referring to the temperament concept proposed by Thomas and Chess (6), children with a “difficult temperament” are at risk for developing conduct problems later in life. For example, Olson et al. (30) found that maternal ratings of “difficult temperament” at 6 months predicted maternal ratings of conduct problems at age 17. Teachers and youth reports on the other hand did not predict these outcomes. Referring to specific temperament dimensions, infant activity level predicts conduct problems during the ages 4–8 (31) as well as during the ages 4–13 (32).

#### Temperament and Attention Deficit Hyperactivity Disorder

Attention deficit hyperactivity disorder (ADHD) is a neurodevelopmental disorder which is characterized by ongoing patterns of inattention and hyperactivity-impulsivity (28). It has been suggested that certain temperament traits are linked to ADHD symptoms (33, 34). For example, low effortful control (EC) is associated with ADHD symptoms in 3–6 year old children [e.g., (35)]. Furthermore, high levels of activity have been shown to predict ADHD-symptoms in preschool aged children (36) as well as school-aged children (37). Additionally, high negative affect can be considered a risk factor for ADHD (35, 37). Further investigating the dimensions of negative affect, Goldsmith et al. (38) found that anger and aggression ratings in kindergarten predicted ADHD symptoms in first graders in school. However, the relation between negative affect and ADHD symptoms might not be due to a direct linkage between these dimensions but rather due to the comorbidity of ADHD with externalizing disorders (33).

#### Temperament as a Protective Factor

Resilience conceptualizes “age-appropriate developmental competences in spite of repeated exposure to biological and psychosocial developmental risk factors” (39). Besides individual, family and contextual influences, temperament can be considered as an additional factor that may promote resilience in children (39). In general, children who display an “easy temperament” (6) show lower levels of behavior problems, higher levels of social competence and higher levels of adaptive behavior (39). With regard to specific temperament traits, positive emotionality is associated with higher levels of social and emotional competence and thus manifests in higher resilience (39). Furthermore, children displaying high levels of approach tendencies show less behavior problems in stressful situations compared to children with low levels of approach tendencies [e.g., (40]. Self-regulation can also be considered a resilience factor. Higher parental ratings of self-regulation are associated with lower levels of internalizing behavior problems (41).

A closer look to the literature reveals a number of shortcomings. Most conclusions are based on cross-sectional research, focusing on only one specific symptom or disorder in a non-clinical sample. Furthermore, not all studies differentiate between temperament dimensions and even fewer consider temperament as a risk and protective factor simultaneously. This study aims at addressing these issues by examining the relation between different dimensions of child temperament and later psychopathological symptoms in a clinical sample.

## Materials and Methods

The study at hand is part of a cohort named “Preschool-Child Development Trajectory Study” with the broad goal of exploring clinical outcomes related to temperament and Emotional Dysregulation (42). We aimed to investigate how child temperament dimensions, assessed at the age of four years, predict later psychopathology at the age of nine years. Participation in the study was voluntary and there was no financial compensation. All children and their parents gave informed consent. The local ethics committee approved the study.

### Participants and Design

**FIGURE 1:**
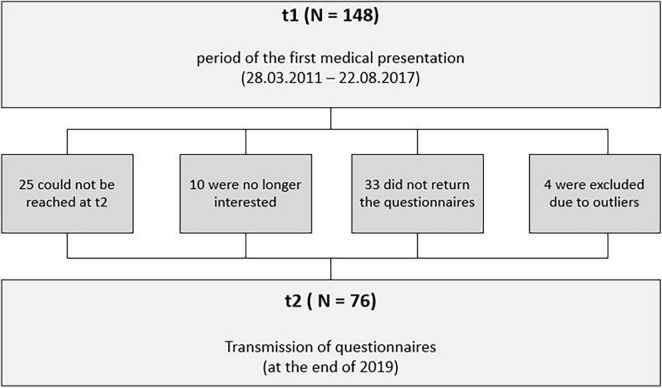
Flow of participants.

In the present study, we used a quasi-experimental design with two measurement points. The original sample included 148 young children who were recruited in 2012–2017 (all young children who had attended the preschool special outpatient clinic; t1) in the child and adolescent psychiatry unit (Figure 1). We are therefore working with a non-representative sample, as it is compiled of a clinical population of patients presented to our outpatient clinic. These families were contacted again at measurement time t2 (at the end of 2019) with a cover letter and a questionnaire. There was no further personal patient presentation at t2. Of the 148 participants, 25 families could not be reached at t2. Ten more families expressed no interest, and 33 families did not return the questionnaires. Of the remaining 80 participants, four were excluded due to outliers in the context of multiple regression, i.e., with more than three standard deviations. Thus, the final sample consisted of *n* = 76 (51% of the original sample). Table 1 shows the sample characteristics. The mean age at t1 was 4.17 years (*SD* = 1.22, 63.2% male, min = 1.33, max = 6.50). 93.4% of the children were diagnosed with at least one disorder according to the ICD-10. The most frequent diagnoses were Oppositional Defiant Disorder (32.89%) and Attention Deficit Hyperactivity Disorder (9.21%). IQ scores were available for 67 children at t1 and were assessed *via* different instruments, mostly WPPSI and SON-R 2$\frac{1}{2}$-7. IQ scores ranged from 50 to 142 (*M* = 100.73, *SD* = 17.58).

**TABLE 1:** Characteristics of sample at t1 (*n* = 76).

Characteristic	*M*	*SD*	Min.	Max.	*n* no medical examination/testing
**Age of child at t1 in years**	**4.17**	**1.22**	**1.33**	**6.50**	
**Body height in cm**	**105.56**	**9.58**	**82.00**	**125.00**	**13**
**Weight child in grams**	**17606.15**	**3548.32**	**10,000**	**26,500**	**11**
**IQ score**	**100.73**	**17.58**	**50**	**142**	**9**
**IQ Test**	***n***	**%**			
None	9	11.84			
WPPSI-III	45	59.21			
SON-R 2$\frac{1}{2}$-7	9	11.84			
KABC-II	9	11.84			
IDS-P	4	5.26			
**Newborn gender**	***n***	**%**			
Male	48	63.16			
Female	28	36.84			
**Main diagnosis**	***n***	**%**			
None	5	6.58			
Dysthymia	1	1.32			
Obsessive-compulsive disorder	1	1.32			
Adjustment disorder	2	2.63			
Non-organic sleep disorders	6	7.89			
Trichotillomania	1	1.32			
Mild mental retardation	1	1.32			
Speech and language development disorders	5	6.58			
Developmental coordination disorder	1	1.32			
Combined specific developmental disorders	1	1.32			
Autism spectrum disorder	2	2.63			
Attention deficit hyperactivity disorder	7	9.21			
Hyperkinetic conduct disorder	1	1.32			
Oppositional defiant disorder	25	32.89			
Other mixed disorders of conduct and emotions	1	1.32			
Phobic anxiety disorder of childhood	2	2.63			
Social anxiety disorder of childhood	1	1.32			
Sibling rivalry disorder	1	1.32			
Other childhood emotional disorders	1	1.32			
Elective mutism	1	1.32			
Chronic motor or vocal tic disorder	1	1.32			
Non-organic encopresis	1	1.32			
Feeding disorder of infancy and childhood	4	5.26			
Stereotyped movement disorders	1	1.32			
Other specified behavioral and emotional disorders with onset usually occurring in childhood and adolescence	2	2.63			
Unspecified behavioral and emotional disorders with onset usually occurring in childhood and adolescence	1	1.32			

*t1, Presentation in child and adolescent psychiatry.*

The mean age at t2 was 9.22 years (*SD* = 2.02, min = 4.67, max = 13). The sample characteristics of t2 are shown in Table 2. At t2, most children attended primary school (50%), followed by high school (19.74%), community schools (11.84%), day care (10.53%) and Waldorf schools (1.32%). Most children (68.42%) lived with their biological parents, 19.74% lived with their biological mother, 1.32% lived with their biological father (and partner), and 2.63% lived in foster care. 14.47% of the children were taking medication, including Methylphenidate (3.95%), or other (10.53%) medication (e.g., Methotrexate, Asthma spray, Melatonin).

**TABLE 2:** Characteristics of sample at t2 (*n* = 76).

Characteristic	*M*	*SD*	Min.	Max.	*n* no medical examination/testing
**Age of child at t2 in years**	**9.22**	**2.02**	**4.67**	**13**	
**Currently visited institution**	***n***	**%**			
Day care center	8	10.53			
Primary school	38	50			
Community school	9	11.84			
Special school	5	6.58			
High school	15	19.74			
Waldorf school	1	1.32			
**Medication**	***n***	**%**			
None	65	85.53			
Methylphenidate	3	3.95			
Other (e.g., Asthma spray, naproxen)	8	10.53			
**Child lives with**	***n***	**%**			
Biological parents	52	68.42			
Biological mother	15	19.74			
Biological mother + partner	2	2.63			
Biological father + partner	1	1.32			
Foster family	4	5.26			
Other	2	2.63			

*t2, Transmission of questionnaires.*

### Instruments and Procedure

#### Assessment of Temperament

We used the Integrative Child Temperament Inventory [ICTI; (43); German version: “Inventar zur integrativen Erfassung des Kind Temperaments”; IKT, (44)] to assess child temperament in preschool aged children at t1. Children between the age of two and eight can be assessed by parents using a six-point Likert scale. The ICTI measures five temperament dimensions using 30 items: activity level (e.g., item 23 “Is constantly moving.”), behavioral inhibition (e.g., item 22 “Is shy when meeting unfamiliar children.”), frustration/anger (e.g., item 25 “Cries or yells when asked to stop favorite occupation”), attention/persistence (e.g., item 28 “Is easily distracted from his/her projects”), and sensory sensitivity (e.g., item 30 “Is sensitive to noise.”). Psychometric properties can be summarized as good (45). Cronbach’s alpha of the five subscales ranges from 0.70 to 0.85 (44). The test-retest reliabilities range from 0.76 to 0.86. The interrater reliability between mothers and fathers ranges from 0.50 to 0.73. Furthermore, convergent and discriminant validities were assessed by comparing the ICTI with the EAS for children (2) and the Child Behavior Questionnaire [CBQ; (46)]. With only one exception (the sensory sensitivity scale showed only a weak convergence to the CBQ scale; *r* = 0.34), these comparisons yielded high correlations.

#### Assessment of Psychopathology

At t2, psychopathology was assessed via the Strengths and Difficulties Questionnaire [SDQ; (15)]. The SDQ is an international screening instrument for assessing various behavioral aspects in children and adolescents aged 2–17 years. It is available free of charge on the internet in over 50 languages. The German parent version of the SDQ was used in the present work. The questionnaire consists of 25 items, which are equally divided into 5 subscales. Four of them are problem scales: emotional symptoms (e.g., item 24 “Many fears, easily scared”), conduct problems (e.g., item 5 “Often has temper tantrums or hot tempers”), hyperactivity/inattention (e.g., item 2 “Restless, overactive, cannot stay still for long”), and peer relationship problems (e.g., item 6 “Rather solitary, tends to play alone”) that can be summed up as one total problem score (total difficulties score). The remaining subscale measures prosocial behavior (e.g., item 1 “Considerate of other people’s feelings”). All items are rated using a three-point Likert scale. For scoring, Goodman used a classification into inconspicuous (80%), borderline (10%), and conspicuous (10%) categories based on a British norming sample. The five scales have been proven by factor analysis in many studies (47–49). The SDQ is a well-suited instrument for assessing children’s problem and prosocial behavior. The psychometric properties of the subscales have mostly satisfactory reliability and validity (15, 50–55).

#### Statistical Analysis

The data was analyzed with IBM SPSS Statistics, version 25. Several multiple linear regression analyses were used to develop a model for predicting SDQ total and subscale scores from temperament traits represented by ICTIs 5 subscales. A significance level of 0.05 was used for all statistical tests and all data were rounded to 2 decimal places. In case of a single missing value in a questionnaire, the rounded individual subscale mean of the respective test was used. This was necessary in 14 cases.

## Results

### Descriptive Analysis

#### Integrative Child Temperament Inventory

Table 3 shows the distribution of the data of the final sample in the ICTI. It is noticeable that most children exceeded the cut off, i.e., are in the 4th quartile, in the subscale activity level (*M* = 23.75, *SD* = 8.21) (39.47%). Almost every third child (32.89%) exceeded the cut off for the subscale frustration (*M* = 22.83, *SD* = 7.18). The values (22.37% and 21.05%) for the subscales behavioral inhibition (*M* = 18.55, *SD* = 6.85) and sensory sensitivity (*M* = 18.22, *SD* = 7.34) were somewhat lower. In between was the subscale attention/persistence (*M* = 20.50, *SD* = 6.59) with 28.95% of conspicuous children.

**TABLE 3:** Descriptive statistics of ICTI (*n* = 76).

	Frustration	Behavioral inhibition	Activity level	Attention/persistence	Sensory sensitivity
*Items*	6	6	6	6	6
*M*	22.83	18.55	23.75	20.50	18.22
*SD*	7.18	6.85	8.21	6.59	7.34
*Minimum*	7	6	7	8	6
*Maximum*	36	36	36	36	34
*Cut-off*	≥75% (Q4)	≥75% (Q4)	≥75% (Q4)	≥75% (Q4)	≥75% (Q4)
*n* > *cut-off*	25	17	30	22	16
*%* > *cut-off*	32.89	22.37	39.47	28.95	21.05

To control the influence of psychopathology at t1, we calculated correlations between temperament factors and internal or external diagnosis. For this purpose, a division into internal (*n* = 20) and external diagnosis (*n* = 38) had to be made. The other cases were excluded from these calculations because they were developmental (*n* = 10), unclassifiable (*n* = 3) or without diagnosis (*n* = 5). There was no correlation between internal/external diagnosis and ICT frustration [*r*_pbis_(56) = 0.21, *p* = 0.11], between internal/external diagnosis and ICT attention/persistence [*r*_pbis_(56) = −0.16, *p* = 0.22] and between internal/external diagnosis and sensory sensitivity [*r*_pbis_(56) = −0.12, *p* = 0.38]. A correlation was found between internal diagnosis and behavioral inhibition [*r*_pbis_(56) = −0.30, *p* = 0.02] and between external diagnosis and activity level [*r*_pbis_(56) = 0.36, *p* = 0.01]. Out of five possible correlations, three were not significant, including frustration with total difficulties score, which is our main finding. Two temperament traits show a correlation that is in line with expectations.

#### Strengths and Difficulties Questionnaire

There were also anomalies found in the subscales and the total difficulties score of the SDQ (Table 4). It stands out that 75% of the children in the subscale peer relationship problems (*M* = 4.26, *SD* = 1.16) exceeded the cut off. In contrast, only 6.58% fell below the cut off in prosocial behavior (*M* = 7.63, *SD* = 1.81). 30.26% were conspicuous with regard to conduct problems (*M* = 2.86, *SD* = 1.25). The values for the subscales emotional symptoms (*M* = 2.16, *SD* = 2.07) and hyperactivity/inattention (*M* = 4.79, *SD* = 1.42) were comparatively low at 15.79% and 14.47%, respectively. Overall, i.e., in relation to the total difficulties score (*M* = 14.07, *SD* = 3.99), 22.37% proved conspicuous.

**TABLE 4:** Descriptive statistics of SDQ parent version (*n* = 76).

	Total difficulties score	Emotional symptoms	Conduct problems	Hyperactivity/Inattention	Peer relationship problems	Prosocial behavior
*Items*	25	5	5	5	5	5
*M*	14.07	2.16	2.86	4.79	4.26	7.63
*SD*	3.99	2.07	1.25	1.42	1.16	1.81
*Minimum*	7	0	1	2	2	3
*Maximum*	25	9	6	8	8	10
*Cut-off (“abnormal”)*	≥17	≥5	≥4	≥7	≥4	≤4
*n* < / > *cut-off*	17	12	23	11	57	5
*%* < / > *cut-off*	22.37	15.79	30.26	14.47	75.00	6.58

### Multiple Regression Analysis

Several multiple regression analysis (method enter) were used to test if child temperament traits of the ICTI and time between t1 and t2 (interim) significantly predict clinical abnormalities in the SDQ. Only the significant models are reported here. However, all results are shown in Table 5.

**TABLE 5:** Multiple Regressions results using ICTI temperament traits and interim (time between t1 and t2) as predictors of SDQ (*n* = 76).

Criterion	Predictor	*B*	*SE B*	β	*T*	*p*	Fit/*R*^2^	*F*	*p*
**SDQ total difficulties score**	ICTI						0.19	2.71	0.02*
	Frustration/anger	0.16	0.08	0.28	2.02	0.047*			
	Behavioral inhibition	–0.13	0.07	–0.23	–1.78	0.08			
	Activity level	–0.05	0.07	–0.09	–0.68	0.50			
	Attention/persistence	–0.10	0.08	–0.17	–1.28	0.21			
	Sensory sensitivity	0.12	0.06	0.22	1.87	0.07			
	Interim	0.00	0.02	0.01	0.06	0.96			
**SDQ emotional symptoms**	ICTI						0.20	2.88	0.02*
	Frustration/anger	0.08	0.04	0.27	1.93	0.06			
	Behavioral inhibition	–0.05	0.04	–0.15	–1.23	0.22			
	Activity level	–0.07	0.03	–0.27	–2.02	0.048*			
	Attention/persistence	–0.05	0.04	–0.16	–1.24	0.22			
	Sensory sensitivity	0.08	0.03	0.30	2.57	0.01*			
	Interim	–0.00	0.01	–0.01	–0.07	0.94			
**SDQ conduct problems**	ICTI						0.13	1.70	0.14
	Frustration/anger	0.04	0.03	0.24	1.64	0.11			
	Behavioral inhibition	–0.00	0.02	–0.02	–0.15	0.88			
	Activity level	0.02	0.02	0.16	1.11	0.27			
	Attention/persistence	–0.01	0.03	–0.03	–0.25	0.81			
	Sensory sensitivity	0.01	0.02	0.04	0.29	0.77			
	Interim	0.00	0.01	0.01	0.04	0.97			
**SDQ hyper-activity** **/inattention**	ICTI						0.17	2.36	0.04*
	Frustration/anger	0.03	0.03	0.16	1.14	0.26			
	Behavioral inhibition	–0.07	0.03	–0.31	–2.45	0.02*			
	Activity level	0.01	0.02	0.08	0.56	0.58			
	Attention/persistence	–0.01	0.03	–0.06	–0.47	0.64			
	Sensory sensitivity	0.03	0.02	0.15	1.29	0.20			
	Interim	–0.01	0.01	–0.09	–0.78	0.44			
**SDQ peer** **relation-ship problems**	ICTI						0.05	0.58	0.74
	Frustration/anger	0.01	0.02	0.04	0.27	0.79			
	Behavioral inhibition	–0.02	0.02	–0.10	–0.69	0.49			
	Activity level	–0.01	0.02	–0.10	–0.66	0.51			
	Attention/persistence	–0.03	0.03	–0.17	–1.23	0.22			
	Sensory sensitivity	–0.00	0.02	–0.01	–0.06	0.95			
	Interim	0.01	0.01	0.14	1.13	0.26			
**SDQ prosocial behavior**	ICTI						0.15	2.06	0.07
	Frustration/anger	–0.04	0.04	–0.17	–1.23	0.23			
	Behavioral inhibition	–0.05	0.03	–0.19	–1.47	0.15			
	Activity level	–0.02	0.03	–0.10	–0.70	0.49			
	Attention/persistence	0.03	0.04	0.10	0.75	0.45			
	Sensory sensitivity	–0.01	0.03	–0.03	–0.25	0.80			
	Interim	0.01	0.01	0.12	1.10	0.28			

B represents unstandardized regression weights, SE B the standard error for B. Beta indicates the standard regression weights. *p < 0.05.

#### Integrative Child Temperament Inventory Temperament Traits Predict Strengths and Difficulties Questionnaire Total Difficulties Score

Regarding SDQ total difficulties score the five ICTI predictors explained a significant proportion of variance [*R*^2^ = 0.19, *F*(6, 69) = 2.71, *p* = 0.02]. It was found that only frustration/anger significantly predicted SDQ total difficulties score [β = 0.28, *t*(69) = 2.02, *p* = 0.047]. Therefore, the final predictive model was: SDQ total difficulties score = 33.83 + (0.16×frustration). The *R*^2^ for the overall model indicates a moderate goodness-of-fit according to Cohen (56), *f*^2^ = 0.49 (strong effect).

#### Integrative Child Temperament Inventory Temperament Traits Predict Strengths and Difficulties Questionnaire Emotional Symptoms

In addition, activity level [β = −0.27, *t*(69) = −2.02, *p* = 0.048] and sensory sensitivity [β = 0.30, *t*(69) = 2.57, *p* = 0.01] could predict SDQ emotional symptoms and explain a significant proportion of variance in SDQ emotional symptoms [*R*^2^ = 0.20, *F*(6,69) = 2.88, *p* = 0.02]. The final predictive model was here accordingly: SDQ emotional symptoms = 7.44 – (0.07 × activity level) + (0.08×sensory sensitivity). The R^2^ for the overall model indicates a moderate goodness-of-fit according to Cohen (56), *f*^2^ = 0.50 (strong effect).

#### Integrative Child Temperament Inventory Temperament Traits Predict Strengths and Difficulties Questionnaire Hyperactivity/Inattention

Finally, behavioral inhibition [β = −0.31, *t*(69) = −2.45, *p* = 0.02] turned out to be a significant predictor for SDQ hyperactivity/inattention accounted for a significant amount of its variance [*R*^2^ = 0.17, *F*(6,69) = 2.72, *p* = 0.04]. The final predictive model here was: 10.07 – (0.07×behavioral inhibition). Following Cohen (56), this is also a strong effect (*f*^2^ = 0.45).

## Discussion

The aim of the present study was to assess the differential impact of child temperament factors on psychopathological outcomes. In a quasi-experimental study with two standardized measures, we assessed temperament factors at age four via the ICTI (43, 44). At age nine, psychopathology was assessed via the SDQ (15). Multiple regression analysis revealed that frustration/anger significantly predicted the total difficulties score in the SDQ. Furthermore, sensory sensitivity served as a significant predictor for emotional symptoms. We also obtained negative correlations between activity level and emotional symptoms as well as between behavioral inhibition and hyperactivity.

The finding, that anger/frustration predicts the total difficulties score in the SDQ tallies with prior work demonstrating that high negative affect is associated with general psychopathology (57). Thus, anger may serve as a risk factor for psychopathological disorders, mood disorders, and spanning personality (58). Further, anger shows longitudinal predictive effects (59) to depression and anxiety disorders (60, 61) and the severity of anger is positively correlated with the intensity of these disorders up to the probability of suicide (62). This finding seems particularly important as it shows that negative affect not only results from psychopathology but can also be considered a precursor for total difficulties score and wide-ranging psychopathology. This result also points out, that temperament (and especially anger/frustration) may be considered a transdiagnostic risk factor for general psychopathology. This proposition is supported by the recent work of Klein et al. (63), who summarize multiple studies indicating irritability to be a transdiagnostic construct. The authors also highlight the prediction of multiple internalizing and externalizing problems within this finding. As they also call for a comparison between irritability and other forms of psychopathology, our approach of using a clinical sample falls in line with their considerations. In line with the finding of temperament being a transdiagnostic risk factor, Ostlund et al. (64) suggested the conceptualization of temperament as a new domain in the Research Domain Criteria (RDoC). Furthermore, the dysregulation of the temperament factor frustration/anger might be considered as part of a larger self-regulatory framework including emotional dysregulation (65). Considering the fact, that poor emotional regulation is also associated with Disruptive Mood Dysregulation Disorder (DMDD) (66), it becomes clear how temperament factors and conduct problems might interact. Sorcher et al. (67) recently investigated the longitudinal associations between irritability in preschool-aged children and adolescent outcomes. They report irritability to predict numerous clinically relevant outcomes, such as anxiety disorders, ADHD and disruptive behavior disorders. They therefore prove irritability to effect peer functionality, physical health and non-suicidal self-Injury. They also show that irritability should be used in large-scale identification due to its incremental and extensive impact. Due to this high impact of anger on general psychopathology, it is important to have the possibility to resort to working anger management treatments. For adults, anger management interventions initiate lasting affective, cognitive, behavioral and physiological changes in reactions of anger (68). For prevention, a variety of interventions like psychotherapy, cognitive-behavioral training, progressive relaxation and skills training (69) have been described as helpful. Moreover, in high-risk children and adolescents, different forms of school-based and individual treatments have been accounted for reducing anger and associated emotional as well as behavioral problems (70–75). The particular position of the temperament factor anger/frustration is also evidenced by the phenomenologically close references to two disorder patterns from the DSM-5, one of which is classified to the domain of behavioral disorders, the other more to the domain of emotional disorders: Oppositional Defiant Disorder (ODD; listed in the DSM-5 under Disruptive, impulse-control, and conduct disorders) and Disruptive Mood Dysregulation Disorder (DMDD; listed in the DSM-5 under mood disorders). However, it will remain a challenge to distinguish between the concepts of temperament and psychopathology (76).

The finding, that sensory sensitivity serves as a risk factor for later emotional symptoms replicates prior work by Ben-Sasson et al. (26) who found, that sensory over-responsivity predicted internalizing, externalizing and dysregulation problems in children at elementary school age. On the other hand, while previous studies identified behavioral inhibition as another predictor for emotional problems [e.g., (77)], behavioral inhibition did *not* predict emotional symptoms in the present study. One reason why this might be the case is that the present study investigated temperamental factors in a clinically referred sample. This is particularly important, because most of the evidence for associations between early child temperament and later psychopathology has been generated in general population studies. Zentner et al. (78) describe four reasons why it is important to examine temperament traits in clinically referred children. First, comparing clinically referred children with general population children allows one to disentangle temperament attributes that might be involved in mild behavior problems from those that are involved in more severe problems. Second, clinically referred children might particularly profit from an understanding of predisposing temperamental factors. Third, identifying temperamental precursors of later psychopathology might give early interventions a better chance to succeed. Fourth, as long as temperament scales cannot differentiate between normally developing children and clinically referred children, their benefits remain limited. While behavioral inhibition did not predict emotional symptoms, it served as a protective factor for hyperactivity in the present study. Identifying protective factors as well as risk factors for later psychopathology is particularly important to identify children at risk for psychopathological outcomes.

Contrary to previous work (36, 37), in the present study activity level did not serve as a risk factor for hyperactivity/inattention. This may also be due to the fact, that the present study investigated the relationship between temperament and psychopathology in a clinically referred sample. While activity level did not predict hyperactivity/inattention in the present study, it served as a protective factor for emotional symptoms. This finding is in line with prior work demonstrating the beneficial impact of activity in the treatment of mood disorders [e.g., (79)]. It might be speculated that increased activity levels lead to more extended exploratory behavior on the part of the child. More exploration behavior, in turn, may be incompatible with avoidance behavior, which is an important maintaining condition for many anxiety disorders. Generally, physical activity is inversely associated with anxiety, depression, and stress reactivity (80).

### Limitations

While the results of the present study give valuable insights into the differential linkage between child temperament and later psychopathology, there are also limitations that should be noted. First, it is important to point out the limited generalizability of our sample, as we are using a non-representative sample, as it is compiled of a clinical population with very heterogeneous diagnoses of patients presented to our outpatient clinic. A clinical sample is biased when it comes to comparing it to the healthy community. It is very well conceivable that specific disorders of the children (especially pervasive developmental disorders) in a specific way and differentially influence the parents’ judgment of the child’s temperament.

A further limitation of the present study is the restriction to parental report measures for the assessment of temperament as well as psychopathology. Questionnaires are subject to a variety of response biases or response errors in the sense of a systematic or unsystematic deviation of the parents’ assessment of the child’s temperament. Parents can certainly assess temperament differently depending on the age of the child. There are certainly additional variables not controlled that have an influence on the assessment of the child’s temperament (e.g., state of the parent-child relationship and attachment, mental disorders of the parents, SES).

Inclusion of other assessment sources such as kindergarten teachers and other methods (laboratory observational measures or structured diagnostic interviews) in the sense of a multi-method and multi-informant approach would put the results on a broader basis.

To control the influence of psychopathology at t1, we calculated correlations between temperament factors and internal or external diagnosis at t1. In summary, out of five possible correlations, three were not significant, including frustration/anger with total difficulties score, which is our main finding. Two temperament traits show a correlation that is in line with expectations. Although the questionnaire used (SDQ) allows standardized statements about the children’s problem behavior at t2, we did not collect any psychiatric diagnoses at t2.

The design of the present study therefore is a quasi-experimental design. To provide further evidence for the results it would be of great value to conduct an *a priori* defined longitudinal study in the future.

## Conclusion

We find that preschool temperament contributes to the development of externalizing and internalizing problems in childhood in a number of ways. The temperament factor frustration/anger in preschool children could be a strong predictor of the general mental condition in 9-year old children. Frustration/anger seems to play a special and prominent role in predicting later general psychopathology. Therefore, it should also play a specific role in the early prevention of emotional dysregulation: high expression of the temperament dimension Frustration/anger should therefore be used as a target for the prevention of psychopathology in children. Activity Level acts as a protective factor against later emotional symptoms. This possibly emphasizes the helpful role of physical activity (for instance, sport) especially in internalizing mental disorders.

This study illustrates the complexity and ambiguity of temperament in child development. What seems to be necessary is the perception of the child’s unique temperament and the child’s unique needs that are dependent on temperament. Temperament in itself is never exclusively positive or negative. It is embedded in the complex and unique development of each person.

## Data Availability Statement

The original contributions presented in the study are included in the article/supplementary material, further inquiries can be directed to the corresponding author/s.

## Ethics Statement

The studies involving human participants were reviewed and approved by the Ethikkommission der Ärztekammer des Saarlandes Ha 147/19. Written informed consent to participate in this study was provided by the participants’ legal guardian/next of kin.

## Author Contributions

FP: idea, conceptualization, structure of the work, data collection, literature analysis, text creation, discussion, and corrections. EM: discussion and corrections. LF: literature analysis, text creation, figure, and tables. JJ: statistical analyses and methods section. All authors contributed to the article and approved the submitted version.

## Conflict of Interest

The authors declare that the research was conducted in the absence of any commercial or financial relationships that could be construed as a potential conflict of interest.

## Publisher’s Note

All claims expressed in this article are solely those of the authors and do not necessarily represent those of their affiliated organizations, or those of the publisher, the editors and the reviewers. Any product that may be evaluated in this article, or claim that may be made by its manufacturer, is not guaranteed or endorsed by the publisher.
